# Randomized controlled trial of social cognition and interaction training compared to befriending group

**DOI:** 10.1111/bjc.12252

**Published:** 2020-06-08

**Authors:** Frances Dark, James G. Scott, Andrea Baker, Stephen Parker, Anne Gordon, Ellie Newman, Victoria Gore‐Jones, Carmen C. W. Lim, Lyndall Jones, David L. Penn

**Affiliations:** ^1^ Metro South Addiction and Mental Health Services, Metro South Addiction and Mental Health Services Princess Alexandra Hospital Woolloongabba Queensland Australia; ^2^ Faculty of Medicine Level 3 UQ Centre for Clinical Research (UQCCR) Herston Queensland Australia; ^3^ Early Psychosis Service Metro North Mental Health Herston Queensland Australia; ^4^ Queensland Centre for Mental Health Research Clinical Support Unit The Park‐Centre for Mental Health Archerfield Queensland Australia; ^5^ Postgraduate Training in Psychiatry, Addiction and Mental Health Services I Metro South Health Blg 23 Garden City Office Park Eight Mile Plains Queensland Australia; ^6^ St Kilda Road Clinic Community Adult Mental Health, Alfred Psychiatry Melbourne Victoria Australia; ^7^ Queensland Brain Institute University of Queensland St Lucia Queensland Australia; ^8^ Pine Rivers Community Health Centre Strathpine Queensland Australia; ^9^ Department of Psychology and Neuroscience University of North Carolina‐Chapel Hill Chapel Hill North Carolina USA; ^10^ Australian Catholic University Fitzroy Victoria Australia

**Keywords:** social cognition, schizophrenia spectrum disorders, randomized controlled trial

## Abstract

**Background:**

Deficits in social cognition are common in people with schizophrenia and are associated with impaired functioning. Finding effective interventions to address these deficits is a priority. Social Cognition Interaction Training (SCIT) is a psychosocial intervention that has demonstrated acceptability and feasibility in various health care settings. Larger, well‐designed randomized controlled trials are needed to examine the effectiveness of this intervention.

**Design:**

A randomized controlled trial.

**Methods:**

One hundred and twenty adults diagnosed with schizophrenia spectrum disorder were randomized to receive SCIT (*n* = 61) or Befriending Therapy (BT) (*n* = 59). Both intervention groups were delivered weekly for 2 hr over 12 weeks. Neurocognitive assessment was completed at baseline. Participants completed assessments of social cognition, social functioning, and meta‐cognition at baseline, post‐intervention, and 3‐month follow‐up.

**Results:**

There were no clinically significant differences between group outcomes on any measure of social cognition or social functioning. There was a trend for both groups to improve over time but not at a level of statistical significance.

**Conclusions:**

SCIT did not show any additional benefits on measures of social cognition compared to Befriending Therapy for people with schizophrenia spectrum disorder. The findings are discussed in terms of potential improvements to the programme.

**Practitioner points:**

- Effective interventions for the social cognitive deficits of schizophrenia spectrum disorders are still being refined.
- Social Cognition Interaction Training is a promising therapy but requires further modifications to improve its effectiveness.

## Background

Deficits in social and community functioning are core features of schizophrenia (Bowie & Harvey, 2005; Green, Kern, Baff, & Mintz, 2000). Pharmacotherapy is not effective for the social cognitive and neurocognitive impairments associated with these poor functional outcomes (Bellack *et al*., 2004; Hogarty, Flesher, Ulrich, Carter, *et al*., 2004; Sergi, Rassovsky, Nuechterlein, & Green, 2006). Thus, psychosocial treatments have been developed to address these impairments. Interventions, such as cognitive remediation, focus on the processes that support or underlie social functioning (i.e., neurocognitive deficits). Although these interventions improve specific domains, their benefits do not automatically generalize to broader psychosocial outcomes (McGurk, Twamley, Sitzer, McHugo, & Mueser, 2007; Wykes, Huddy, Cellard, McGurk, & Czobor, 2011). This has led to the investigation of additional factors that contribute to functional impairments such as social cognition. Social cognition encapsulates cognitive processes involved in the recognition, understanding, accurate processing, and effective use of social cues in real‐world situations (Green *et al*., 2008). Social cognition is considered to have four domains: emotional and social perception, theory of mind (ToM), and attributional style (Pinkham *et al*., 2014). There is growing evidence that social cognition is an independent predictor of function but also mediates the relationship between neurocognition and functional outcomes in schizophrenia (Fett *et al*., 2011).

A number of therapies have arisen to address the multiple domains of social cognition. The first meta‐analysis of therapeutic approaches found large effect sizes for emotional recognition (=0.71), moderate effect sizes for theory of mind (*d* = 0.46), and large effect sizes for observed improvements in social functioning (*d* = 0.78) (Kurtz & Richardson, 2012). A more recent meta‐analysis screened for study methodology and largely supported these findings but with notable caveats especially about suboptimal study methodology. Social Cognition and Interaction Training (SCIT) (Roberts, Penn, & Combs, 2016) was the most studied multidomain intervention in these meta‐analyses (Grant, Lawrence, Preti, Wykes, & Cella, 2017; Kurtz & Richardson, 2012).

SCIT is a group‐based therapy that comprises three phases (‘Introduction & Emotions’, ‘Figuring out Situations’, and ‘Checking it out’). A series of open trials have demonstrated that SCIT can be implemented in a variety of settings (inpatient and outpatient), is generally well‐received by clients and therapists, and is associated with improvements in social cognition and social functioning among individuals with schizophrenia (Combs *et al*., 2007; Roberts & Penn, 2009). To date, there have been only a small number of randomized controlled trials of SCIT (Gordon *et al*., 2017; Hasson‐Ohayon, Mashiach‐Eizenberg, Avidan, Roberts, & Roe, 2014; Roberts *et al*., 2014; Tas, Danaci, Cubukcuoglu, & Brune, 2012; Taylor *et al*., 2016; Wang *et al*., 2013). The results from these trials have been less uniform with Gordon et.al in a waitlist controlled design (*n* = 36) reporting no significant improvement in any measure but there was high acceptability of SCIT with around 86% of participants completing the programme. Roberts et al. in a medium‐sized (*n* = 66) randomized controlled trial of SCIT compared with treatment as usual found no significant improvement in the intervention group on measures of social cognition; however, there was some suggestion that those receiving SCIT did better on measures of functioning and negative symptoms. Larger trials addressing some of the methodological issues raised in the preceding studies are needed to further evaluate the effectiveness of SCIT. (Grant *et al*., 2017; Kurtz, Gagen, Rocha, Machado, & Penn, 2016).

### Objectives

This randomized, controlled trial aimed to compare the effectiveness of SCIT for individuals with schizophrenia spectrum disorders with an active control group, Befriending Therapy (BT). It was hypothesized that individuals who receive SCIT would show greater improvement in social cognition as measured by the Bell Lysaker Emotion Recognition Task (BLERT) (Bryson, Bell, & Lysaker, 1997); Internal, Personal, and Situational Attributions Questionnaire (IPSAQ) (Kinderman & Bentall, 1996); Hinting Task (HT) (Corcoran, Mercer, & Frith, 1995); and social functioning as measured by the Social Functioning Scale (SFS) (Birchwood, Smith, Cochrane, Wetton, & Copestake, 1990) and the Social Skills Performance Assessment (SSPA) (Patterson, Moscona, McKibbin, Davidson, & Jeste, 2001) compared to individuals receiving BT at endpoint (week 12).

## Method

### Participants

**Figure 1:**
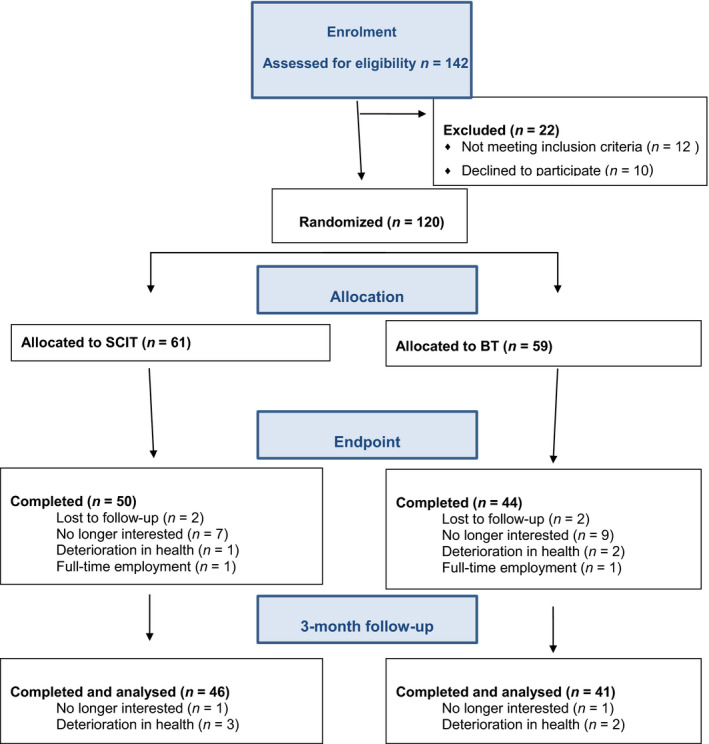
CONSORT flow diagram. [Colour figure can be viewed at wileyonlinelibrary.com]

One hundred and forty‐two people attending community mental health clinics in Brisbane, Australia, were screened and twenty‐two excluded from the study (Figure 1). One hundred and twenty (120) eligible participants provided written consent and were randomized to either SCIT or BT. Participants met the Diagnostic and Statistical Manual of Mental Disorders 5^th^ Edition (DSM 5) criteria for schizophrenia spectrum disorders based on the Diagnostic Interview for Psychosis (DIP) (Castle *et al*., 2006). They were between 18 and 65 years of age and had interpersonal communication difficulties based on a score of < 105 on the Interpersonal Communication Subscale Score of the Social Functioning Scale (Birchwood *et al*., 1990).

### Intervention delivery

SCIT is a manualized group therapy of 20–24 hourly sessions (Roberts *et al*., 2016). In this study, a weekly session covered the equivalent of two sessions from the manual. Each session lasted 2 hr. The total programme duration was 12 weeks. Delivery in this format was intended to address attrition issues observed in an earlier pilot study (Parker *et al*., 2013) and is consistent with alternate delivery formats described in the SCIT manual (Roberts *et al*., 2016). Groups were based on a maximum of 8 participants per group with 2 facilitators. All clinicians had received 1‐day face‐to‐face training by the principal investigator (FD) who was endorsed by the programme developers (DP and DR) to train in SCIT. Less experienced staff co‐facilitated with experienced staff, and discussion about adherence to the manual occurred at the end of each session.

BT was developed as a control intervention for clinical trials of psychosocial interventions (Bendall *et al*., 2006). BT has been found to have a small effect in reducing symptoms in people with psychosis (Sensky *et al*., 2000). It is matched in many respects to SCIT (e.g., group format, duration, frequency), but does not include social cognitive skills development. It is comprised of a series of group conversations focusing on neutral, non‐clinical topics of interest to the participants. The facilitators of BT were trained by the principal investigator (FD). They were predominately peer workers employed by the mental health services who had experience conducting group work. One group was co‐facilitated by a social worker.

All sessions, of both interventions, were audio‐recorded, and these recordings were randomly assessed for adherence and fidelity by one of the investigators. The sessions were reviewed by experienced therapists who had not conducted the groups they were assessing (FD and SP) with feedback given to group facilitators to minimize any deviation from either manual (SCIT or BT).

### Study assessments and procedures

The study commenced in June 2016, and recruitment was completed by November 2018. An independent member of the research team not involved in the delivery of the intervention or outcome assessment was emailed the list of participants for randomization. A computer‐generated randomization list of random numbers was created using SAS 9.4 (SAS Institute, NC, USA) with a 1:1 allocation using block sizes of 4.

All participants in the study continued to receive standard clinical care (i.e., there were no restrictions on medication or psychosocial interventions, apart from participants receiving SCIT or BT in addition to the standard treatments).

Trained research assistants, who were blind to group allocation, consented participants and conducted assessments at baseline, 12 weeks, and 12 weeks from programme completion.

To minimize dropout rates, participants were given reminder calls the day before group; followed up immediately with a phone call if the participant did not attend the group; and providing transportation (if necessary) to group.

Participants were reimbursed for out‐of‐pocket expenses, inconvenience, and time involved by the provision of $50 prepaid gift cards on completion of the baseline assessment, end‐of‐study assessment, and follow‐up assessment 3 months later (total reimbursement $150).

### Measures

#### Social cognition

Emotional perception was assessed using the Bell Lysaker Emotion Recognition Task (BLERT), which measures the ability to correctly identify seven emotional states: happiness, sadness, fear, disgust, surprise, anger, or no emotion (Bryson *et al*., 1997). The BLERT is scored out of 21 with higher scores indicating better social cognition.

Theory of mind (ToM) was assessed using the Hinting Task (HT) (Corcoran *et al*., 1995). The HT consists of ten short stories that involve an interaction between two characters, one of which drops a hint at the end of the story. The participant**’**s task is to infer what the character really means by their hint. Higher scores indicate better ToM with the maximum score being 20.

Attribution bias was assessed using the Internal, Personal, and Situational Attributions Questionnaire (IPSAQ) (Kinderman & Bentall, 1996). The IPSAQ is used to assess attribution style. This measure consists of 32 items that describe in second person 16 positive and negative social situations. Two derivative scores are produced: ‘externalizing bias’ and ‘personalizing bias’. For both domains, scores range from 0 to 1.

#### Social functioning

Social functioning was measured using the Social Functioning Scale (SFS) (Birchwood *et al*., 1990) and the Social Skills Performance Assessment (SSPA) (Patterson *et al*., 2001). The SFS is a 79‐item questionnaire with seven domains. The raw score was transformed using a standardized distribution with a mean of 100 and standard deviation of 15. The SFS has been found to have good reliability (test–retest, and informant/self‐report reliability, item‐total correlation) and validity (construct, concurrent, and criterion group validity) (Birchwood *et al*., 1990). A high score indicates a high level of functioning. The SSPA assesses social functioning in which the subject participates in three role‐plays presenting selected social problem situations. All role‐plays were videotaped to enable inter‐rater reliability analysis. Scores were rated on a Likert‐type scale from 1 to 5 where 5 indicates little or no deficiency. There are 17 categories in total. Scores from each role‐plays were summed with total scores ranging from 17 to 85. The SSPA has good reliability (test–retest of 0.92) and inter‐rater reliability (intraclass correlation of .91) (Patterson *et al*., 2001).

#### Neurocognition

Neurocognition was assessed using the CogState Brief Battery (CBB), which is a computer‐administered cognitive test battery that consists of cognitive tasks that measure psychomotor function, attention, and working memory and has good psychometric properties (Maruff *et al*., 2009). This was assessed at baseline to enable assessment of whether neurocognitive abilities impacted outcomes.

#### Meta‐cognition

Meta‐cognition, which has been hypothesized as a mechanism of skills transfer in psychosocial interventions, was measured using the Meta‐Cognition Questionnaire (MCQ‐30), which consists of 30 items measuring an individual**’**s meta‐cognitive beliefs, judgements, and monitoring (Wells & Cartwright‐Hatton, 2004). The MCQ‐30 has 30 items with 5 factors reflecting the dimensions of meta‐cognition. The maximum score is 24, and the total score for the task is 120.

#### Endpoints

The primary outcome was improvement in Bell Lysaker Emotion Recognition Task (BLERT). Secondary outcomes were improvement in SFS, SSPA, HT, and IPSAQ at endpoint (12 weeks). Co‐secondary outcomes were the persistence of effects in SFS, SSPA, HT, BLERT, and IPSAQ at the 24‐week follow‐up assessment (12 weeks post‐end of SCIT).

#### Sample size

We calculated our sample size based on our primary outcome, emotional recognition assessed using BLERT. The choice of primary outcome measure was based on (1) the first meta‐analysis of social cognitive interventions revealing large effect sizes for emotional recognition (Kurtz *et al*., 2015) and the finding that emotional recognition is associated with social functioning (Javed & Charles, 2018). We estimated sample size based on two sources: (1) previous research that has investigated the effects of psychosocial treatment on emotional perception (Silver, Goodman, Knoll, & Isakov, 2004) and (2) the results of open‐trial pilot studies (effect sizes ranging from 0.6 to 1.6) (Roberts, Penn, Labate, Margolis, & Sterne, 2010). Based on a conservative effect size of 0.84, and a baseline–post‐intervention correlation of 0.5, a sample of 28 participants per group (*n* = 56) was required to achieve 80% power with the alpha level set at .05. Assuming a dropout rate of 20%, a minimum sample of approximately 70 participants was required, thus 35 participants in each group. Due to practicalities of group size and opportunities for running the groups over the study time frame, we randomized 120 participants.

### Statistical method

Analysis on the primary and secondary outcomes was performed based on the intention‐to‐treat (ITT) sample (i.e., participants analysed according to randomized treatment assignment ignoring non‐compliance and dropouts). The primary outcome was analysed using linear mixed models to estimate the effect of the intervention on emotional recognition from baseline to post‐intervention (endpoint) and from baseline to 3‐month follow‐up. Treatment group (a binary indicator for the intervention group (SCIT/BT)) and baseline BLERT scores were included as fixed effects, while site was included as random effects in the mixed model. Since participants were recruited across small and large sites with potential variation between sites in the severity of the illness of people recruited, it was desirable to control for site‐effects in the RCT analysis (Kahan, 2014). A similar modelling approach was undertaken on all secondary outcomes (MCQ scores, HT scores, etc.). Least squares mean and standard error along with difference in least squares means between groups were reported. Approximately two thirds of the participants completed at least six sessions. Post‐hoc analyses were carried out on participants who attended at least 12 out of 20 sessions with no major protocol violations and in the subsample of people with schizophrenia. Effect sizes based on Cohen’s *d* for all the outcomes were also calculated. Significance level was evaluated at the .05 level using 2‐sided test. All analysis was conducted using SAS software, version 9.4.

## Results

The flow diagram detailing recruitment and withdrawal from treatment throughout the study is presented in Figure 1.

The groups did not significantly differ in any demographic variables or baseline neurocognitive variables (Table 1) or on baseline measures of social cognition and social functioning (Table 2). Ninety‐five (82%) of participants had a diagnosis of schizophrenia. All patients were prescribed antipsychotic medication; the majority were on clozapine, aripiprazole, olanzapine, and paliperidone (see Appendix 1). SCIT participants attended an average of 6.3 (*SD* = 3.8) out of the twelve available sessions. The BT participants attended a mean of 6.6 (*SD* = 4.1) sessions.

**Table 1:** Baseline demographic and cognitive characteristics by the intervention group

Characteristics	BT (*n* = 59)	SCIT (*n* = 61)	Total (*n* = 120)	Test statistic, *p‐*value
Age in years – Mean (*SD*)	37.5 (10.1)	36.1 (10.7)	36.8 (10.4)	*t* = 0.71, *p* = .477
Males – No. (%)	40 (67.8)	46 (75.4)	86 (71.7)	χ^2^ = 0.86, *p* = .354
Aboriginal/Torres Strait Islander – No. (%)	3 (5.1)	5 (8.2)	8 (6.7)	χ^2^ = 0.47, *p* = .494
Country of Birth – No. (%)				
Australia	44 (74.6)	45 (73.8)	89 (74.2)	χ^2^ = 0.01, *p* = .920
Employment – No. (%)				
Part‐time	5 (8.4)	1 (1.6)	6 (5.0)	χ^2^ = 3.25, *p* = .354
Casual	2 (3.4)	2 (3.3)	4 (3.3)	
Unemployed	4 (6.8)	3 (4.9)	7 (5.8)	
Government benefit	48 (81.4)	55 (90.2)	103 (85.8)	
Living situation – No. (%)				
Independent (alone)	17 (28.8)	18 (29.5)	35 (29.2)	χ^2^ = 2.56, *p* = .279
Family	9 (15.2)	16 (26.2)	25 (20.8)	
Supported housing	33 (55.9)	27 (44.3)	60 (50.0)	
Cognitive state battery^a^ – mean (*SD*)				
Detection task (DET)^b^	2.5 (0.4)	2.6 (0.1)	2.5 (0.3)	*t* = −0.9, *p* = .394
Identification task (IDN)^c^	2.7 (0.1)	2.7 (0.1)	2.7 (0.1)	*t* = −0.5, *p* = .591
One Card Learning (OCL)^d^	0.9 (0.2)	0.9 (0.1)	0.9 (0.1)	*t* = −0.6, *p = *.547
One Back task (ONB)^e^	2.9 (0.1)	2.9 (0.1)	2.9 (0.1)	*t* = −1.3, *p = *.182

Notes

Higher score indicates better performance.

^a^ The cognitive state battery is a computerized series of tasks which uses playing cards to test aspects of cognition. The tasks included in this study were DET, IDN, OCL, and ONB

^b^ DET assesses processing speed in log_10_ milliseconds. Lower score indicates better performance

^c^ IDN assesses visual learning and memory in log_10_ milliseconds. Lower score indicates better performance

^d^ OCL assesses the accuracy of the performance in the form of arcsine square root of the proportion of correct responses

^e^ ONB assesses attention/working memory in log_10_ milliseconds. Lower score indicates better performance.

**Table 2:** Baseline measures of social cognition by the intervention group

Outcomes	Mean (*SD*)			Test statistic, *p‐*value	Effect size, Cohen’s *d*
	BT (*n* = 59)	SCIT (*n* = 61)	Total (*n* = 120)		
I. BLERT					
Total score	14.1 (4.0)	14.7 (3.1)	14.4 (3.6)	*t* = −0.95, *p* = .343	0.17
II. Social Functioning Scale (SFS)					
Social withdrawal	98.6 (7.8)	99.4 (9.4)	99.0 (8.6)	*t* = −0.53, *p* = .600	0.09
Interpersonal communication	94.3 (11.3)	96.8 (9.9)	95.6 (10.6)	*t* = −1.27, *p* = .206	0.24
Independence performance	108.7 (10.2)	105.1 (11.4)	106.9 (10.9)	*t* = 1.81, *p* = .073	0.34
Recreation activities	108.8 (13.8)	104.9 (15.3)	106.8 (14.6)	*t* = 1.45, *p* = .151	0.27
Pro‐social activities	100.1 (12.9)	100.7 (12.8)	100.4 (12.8)	*t* = −0.26, *p* = .797	0.05
Independence competence	97.8 (13.8)	94.0 (14.4)	95.8 (14.2)	*t* = 1.48, *p* = .141	0.27
Occupational/employment	101.1 (9.4)	99.3 (10.4)	100.2 (9.9)	*t* = 0.96, *p* = .337	0.18
Total score	101.3 (7.4)	100.0 (7.5)	100.7 (7.5)	*t* = 0.95, *p* = .344	0.18
III. Meta‐Cognition Questionnaire (MCQ)					
Total score	68.0 (16.6)	67.4 (12.8)	67.7 (14.7)	*t* = 0.23, *p* = .818	0.04
IV. Hinting Task (HT)					
Total score	15.6 (3.4)	15.8 (3.3)	15.7 (3.4)	*t* = −0.39, *p* = .694	0.06
V. Internal, Personal, and Situational Attributions Questionnaire (IPSAQ)					
Externalizing bias	1.4 (3.4)	1.2 (3.7)	1.3 (3.5)	*t* = 0.35, *p* = .726	0.06
Personalizing bias	1.0 (0.8)	0.9 (0.6)	0.9 (0.7)	*t* = 0.80, *p* = .428	0.14
VI. Social Skills Performance Assessment (SSPA)					
Scenario 1	30.6 (5.5)	29.9 (5.2)	30.2 (5.4)	*t* = 0.73, *p* = .469	0.13
Scenario 2	35.6 (5.4)	35.0 (6.0)	35.3 (5.8)	*t* = 0.60, *p* = .550	0.11
Total score	66.2 (10.2)	64.9 (10.4)	65.5 (10.3)	*t* = 0.71, *p* = .478	0.13

### Social cognition and social functioning outcomes

The main effects analyses showed at endpoint (week 12), and SCIT participants did not differ significantly from BT participants in terms of emotional recognition (*p* = .185) although there was a small effect size in favour of SCIT (ES = −0.18). SCIT participants did not differ significantly from BT participants on any other measures of social cognition or social functioning (Table 3). Similarly, at 3‐month post‐intervention follow‐up, there were no significant differences between both groups of participants across all outcomes (Table 4).

**Table 3:** Estimated treatment effect at post‐treatment (week 12)

Outcomes	Post‐intervention, least squares means (*SE*)		Between‐condition differences		
	BT	SCIT	Differences of least squares means (*SE*)^a^	*p*‐value	Effect size, Cohen’s *d*
I. BLERT					
Total score	14.7 (0.7)	15.5 (0.6)	0.8 (0.6)	.185	−0.18
II. Social Functioning Scale (SFS)					
Social withdrawal	101.0 (1.3)	101.5 (1.1)	0.5 (1.7)	.776	−0.06
Interpersonal communication	109.3 (2.8)	105.3 (2.5)	−4.0 (3.7)	.294	0.22
Independence performance	107.2 (1.5)	108.9 (1.3)	1.8 (2.0)	.384	−0.18
Recreation activities	103.6 (2.0)	104.2 (1.8)	0.6 (2.6)	.807	−0.05
Pro‐social activities	100.9 (1.7)	103.3 (1.5)	2.4 (2.3)	.308	−0.22
Independence competence	96.6 (2.4)	97.9 (2.2)	1.3 (2.8)	.649	−0.08
Occupational/employment	99.8 (1.3)	100.3 (1.2)	0.5 (1.7)	.753	−0.06
Total score	102.7 (1.2)	102.9 (1.1)	0.3 (1.6)	.852	−0.03
III. Meta‐Cognition Questionnaire (MCQ)					
Total score	66.7 (1.7)	64.2 (1.6)	−2.5 (2.3)	.295	0.22
IV. Hinting Task (HT)					
Total score	17.0 (0.4)	16.9 (0.4)	−0.1 (0.5)	.844	0.04
V. Internal, Personal, and Situational Attributions Questionnaire (IPSAQ)					
Externalizing bias	1.8 (0.4)	1.9 (0.4)	0.1 (0.3)	.993	−0.04
Personalizing bias	0.9 (0.1)	0.7 (0.1)	−0.1 (0.1)	.182	0.29
VI. Social Skills Performance Assessment (SSPA)					
Scenario 1	32.5 (0.8)	32.1 (0.8)	−0.4 (1.1)	.703	0.07
Scenario 2	36.5 (0.8)	35.9 (0.7)	−0.6 (1.1)	.595	0.12
Total score	68.9 (1.5)	67.9 (1.5)	−0.9 (2.0)	.647	0.10

Notes

BT = Befriending Therapy; SCIT = Social Cognition Interaction Training; *SE* = standard error.

^a^ The reference group is BT.

**Table 4:** Estimated treatment effect at 3‐month follow‐up

Outcomes	3‐month post‐intervention, least squares mean (*SE*)		Between‐condition differences		
	BT	SCIT	Differences of least squares means (*SE*)^a^	*p*‐value	Effect size, Cohen’s *d*
I. BLERT					
Total score	15.1 (0.5)	15.3 (0.5)	0.2 (0.6)	.185	−0.06
II. Social Functioning Scale (SFS)					
Social withdrawal	101.0 (1.4)	103.2 (1.5)	2.2 (2.0)	.289	−0.23
Interpersonal communication	112.8 (2.7)	110.4 (2.8)	−2.4 (3.6)	.506	0.13
Independence performance	108.7 (1.5)	110.7 (1.6)	1.9 (1.8)	.307	−0.20
Recreation activities	109.2 (2.2)	108.2 (2.3)	−1.0 (2.9)	.747	0.07
Pro‐social activities	105.6 (2.1)	103.5 (2.2)	−2.1 (2.5)	.418	0.15
Independence competence	98.1 (2.7)	99.7 (2.8)	1.6 (2.8)	.573	−0.09
Occupational/employment	100.6 (1.2)	102.0 (1.3)	1.4 (1.7)	.426	−0.17
Total score	105.0 (1.5)	105.4 (1.5)	0.4 (1.6)	.809	−0.04
III. Meta‐Cognition Questionnaire (MCQ)					
Total score	65.2 (1.7)	62.1(1.8)	−3.1 (2.3)	.203	0.27
IV. Hinting Task (HT)					
Total score	17.6 (0.4)	17.1 (0.4)	−0.5 (0.5)	.352	0.19
V. Internal, Personal, and Situational Attributions Questionnaire (IPSAQ)					
Externalizing bias	1.0 (0.6)	1.1 (0.5)	0.1 (0.7)	.905	−0.03
Personalizing bias	0.9 (0.1)	0.9 (0.1)	0.0 (0.1)	.967	0.00
VI. Social Skills Performance Assessment (SSPA)					
Scenario 1	32.9 (0.6)	33.2 (0.6)	0.3 (0.8)	.689	−0.08
Scenario 2	37.2 (0.6)	37.1 (0.7)	−0.1 (0.9)	.896	0.02
Total score	70.1 (1.2)	70.3 (1.2)	0.2 (1.5)	.896	−0.03

Notes

BT = Befriending Therapy; SCIT = Social Cognition Interaction Training; *SE* = standard error.

^a^ The reference group is BT.

To observe whether the effect of intervention on emotional recognition was maintained at 3 months post‐intervention, we examined a group x time interaction model where time was treated as a categorical variable assuming an unstructured covariance. There was no significant time x treatment group interaction (*F* = 1.39, *p* = .252), which indicates that there was no difference in patterns of change between both groups over time. The time effect was significant (*F* = 3.07, *p* = .049) with both treatments showing some evidence of improvement over time from baseline to endpoint, and the effect was maintained at the 3‐month follow‐up.

### Post‐hoc analysis of participants completing the programmes

We conducted a post‐hoc analysis on a subset of participants who completed at least 6 weeks (12 sessions) of the programme (*n* = 73). There were 34 SCIT and 39 BT participants. This subgroup of participants did not differ from the broader sample in terms of demographic, cognitive state and primary outcome (emotional recognition). The results of the post‐hoc analyses showed no significant difference in social cognition or functional outcomes between these groups at any time period in the subgroup of participants who completed at least 6 weeks of the programme (see Appendix 1).

### Post‐hoc analysis of people with schizophrenia

Our study has recruited participants with schizophrenia spectrum disorders (a cohort which is more heterogeneous in terms of the schizophrenia diagnosis). We also conducted a post‐hoc analysis on a subset of people with schizophrenia. Again, this subgroup of participants did not differ in terms of the primary outcome, total BLERT scores at endpoint (week 12), and at 3‐month post‐intervention follow‐up (week 24) (see Appendices 3 and 4).

### Meta‐cognition

There was no significant difference between both groups on total MCQ score.

## Discussion

This large randomized controlled trial found no significant difference between SCIT and BT group participants on social cognition and functional outcome measures. This finding is not consistent with a recent meta‐analysis of approaches to remediate social cognition which found these interventions to be associated with improvement in affect recognition and theory of mind in studies with active and passive controls (Grant *et al*., 2017). There are a number of possible explanations for the lack of a significant improvement in social cognition in the SCIT participants compared to the BT participants. The participants were consumers of an Australian public mental health service. Most participants had severe and long‐standing schizophrenia, which was refractory to other interventions. Two hours per week for 12 weeks may not be enough to result in any meaningful change in this population. Session adherence was on average around 50%, which is lower than that from trials in Israel recruiting participants from rehabilitation services where the mean session attendance was 71% ± 24%. In our previous pilot studies, we found lower adherence for participants recruited from community settings (Parker, Foley, Walker, & Dark, 2013). In addition, despite a power analysis being conducted, the study may still not have been powered to show small effects. The developers of SCIT have previously suggested a threshold of 50% of sessions attended as an estimate of adequate treatment (Roberts *et al*., 2014; Roberts & Penn, 2009). Roberts et al. suggest a possible dose–response effect may exist in SCIT therapy and recommends more frequent and intense sessions may be useful (Roberts *et al*., 2014).

Consistent with our pilot work (Parker *et al*., 2013), attrition remained a significant concern especially for participants residing independently in the community. Even with attention to ensuring participant retention, one quarter of participants in both groups did not receive the allocated intervention. In providing psychosocial interventions to people living with schizophrenia, it is important to consider whether the participants can commit to the length of the programme. Further consistent efforts are required by staff to keep participant motivation high and to address any potential barriers to attendance. In the early stages of therapy, assistance to get to sessions can aid in encoding the value of attendance.

The heterogeneity of the population diagnosed with schizophrenia spectrum disorders can confound results. In this study, we did screen to ensure participants recruited did have social functioning deficits. Roberts et al. have also suggested that SCIT may be particularly suited to the subset of people diagnosed with schizophrenia who have dysfunctional attribution biases (Roberts *et al*., 2014).

Interventions to address social cognition are in their infancy. SCIT targets multiple social cognition domains with the associated challenge of ensuring each domain receives the correct ‘dose’ of treatment. Focusing on a single social cognitive domain may enable more precise selection of participants with that deficit. This would be consistent with current modular approaches to cognitive behaviour therapy for psychosis (Addington & Gleeson, 2018; Kowalski, Pankowski, Lew‐Starowicz, & Gawęda, 2017; Steel, 2008). Roberts has recently extracted parts of the ‘figuring it out’ phase of SCIT to deliver as a stand‐alone module to target attribution bias (Roberts, Kleinlein, & Stevens, 2012). Further refinement is required to the SCIT intervention when delivered to this population in order to confer a benefit. This may involve modifying the selection of participants, the dose of intervention, or the actual intervention.

### Strengths and limitations

The strengths of this study include the successful completion of an adequately powered intervention to people with severe illness attending a community mental health service delivered within existing resources.

Limitations included recruiting from residential rehabilitation centres where the participants may have discussed the interventions with each other potentially diluting any difference in the programmes. No standardized symptom ratings were conducted, which may have limited the ability to detect benefits on non‐targeted symptom domains. It is known that most psychosocial interventions, including SCIT, are recommended to be delivered when patients are in a relatively stable phase of their illness (Hogarty, Flesher, Ulrich, & *et al*., 2004; Medalia & Richardson, 2005), with no standardized measures of symptomatology and the assumption that living in the community equated with relative mental stability may have resulted in participants attending at a time when they were too symptomatic to optimally benefit.

Additionally, participant programme satisfaction ratings were not conducted. These data could have informed future programme iterations.

### Recommendations for future research

Ongoing research to develop interventions to address the social cognitive impairments associated with schizophrenia should be a priority given the relationship between these symptoms and functional outcomes. It is important that these findings and recommendations from RCTs are incorporated into the implementation of SCIT in mental health services (Dark *et al*., 2016). Future research needs to be conducted on the effectiveness of SCIT in patients who are assessed to have a high level of paranoia (Kowalski *et al*., 2017). Roberts has suggested increasing the frequency of the sessions to twice weekly (Roberts *et al*., 2014). Motivational principles such as linking session topics to personal goals, and frequent reinforcement for participation should be used to maximize attendance to increase the dose received by participants. Sex differences have been reported in social cognition (Zhang et al., 2017). Future studies with larger sample sizes should examine whether there are sex differences in the response to SCIT. Given the stepped nature of the SCIT programme, a study where participants only progress through modules after achieving minimal competency in each phase may ensure that the intervention is delivered at the correct dose for each individual.

### Conclusion

There is a very high level of disability experienced by many living with schizophrenia. To date, pharmacotherapy has been ineffective for social cognitive and neurocognitive impairments. It is essential that researchers and clinicians continue to collaborate in order to develop and evaluate psychosocial interventions, which aim to improve the lives of people living with schizophrenia.

## Author contributions

**Frances Dark** (Conceptualization; Funding acquisition; Methodology; Writing – original draft; Writing – review & editing) **James Scott** (Formal analysis; Supervision; Writing – review & editing) **Andrea Baker** (Data curation; Project administration; Supervision) **Stephen Parker** (Conceptualization; Formal analysis; Writing – review & editing) **Anne Gordon** (Conceptualization; Data curation; Writing – review & editing) **Ellie Newman** (Data curation; Methodology; Project administration; Writing – review & editing) **Victoria Gore‐Jones** (Investigation; Writing – review & editing) Carmen Lim (Formal analysis; Methodology; Writing – original draft; Writing – review & editing) **Lyndall Jones** (Conceptualization; Project administration; Writing – review & editing) **David Penn** (Conceptualization; Formal analysis; Methodology; Project administration; Supervision; Writing – original draft; Writing – review & editing).

## Conflicts of interest

DP is a co‐developer of SCIT. The other authors have no conflict of interest.

## Ethical approval

The Metro South Human Research Ethics Committee provided ethical clearance for this study (HREC/16/QPAH/98).

## Data Availability

De‐identified data are available from the corresponding author.

## Appendix

**Table A1:** Estimated treatment effect at post‐treatment (week 12) among those that completed at least 6 sessions (*n* = 70)

Outcomes	Post‐intervention outcome, least squares mean (*SE*)		Between‐condition differences		
	BT	SCIT	Differences of least squares means (*SE*)^a^	*p*‐value	Effect size, Cohen’s *d*
I. BLERT					
Total score	14.4 (1.0)	15.2 (1.0)	0.8 (0.6)	.199	−0.07
II. Social Functioning Scale (SFS)					
Social withdrawal	100.8 (1.4)	101.9 (1.5)	1.1 (2.0)	.582	−0.13
Interpersonal communication	111.3 (3.0)	103.1 (3.0)	−8.1 (4.3)	.068	0.47
Independence performance	106.7 (1.5)	110.6 (1.6)	3.9 (2.3)	.112	−0.43
Recreation activities	103.7 (2.1)	105.8 (2.2)	2.1 (3.0)	.480	−0.17
Pro‐social activities	100.2 (2.1)	103.2 (2.2)	2.9 (2.6)	.269	−0.24
Independence competence	96.2 (2.4)	100.6 (2.4)	4.5 (3.3)	.188	−0.31
Occupational/employment	100.1 (1.3)	100.9 (1.4)	0.8 (1.9)	.683	−0.10
Total score	102.7 (1.3)	103.7 (1.3)	1.0 (1.8)	.575	−0.13
III. Meta‐Cognition Questionnaire (MCQ)					
Total score	63.7 (1.8)	61.3 (1.8)	−2.3 (2.6)	.376	0.23
IV. Hinting Task (HT)					
Total score	17.0 (0.5)	16.7 (0.5)	−0.3 (0.7)	.645	0.10
V. Internal, Personal, and Situational Attributions Questionnaire (IPSAQ)					
Externalizing bias	1.8 (0.5)	2.2 (0.6)	0.4 (0.3)	.575	−0.12
Personalizing bias	0.9 (0.1)	0.8 (0.1)	−0.1 (0.1)	.321	0.17
VI. Social Skills Performance Assessment (SSPA)					
Scenario 1	32.4 (0.9)	32.3 (0.9)	−0.1 (1.3)	.917	0.02
Scenario 2	36.5 (0.9)	35.6 (0.9)	−0.9 (1.3)	.498	0.17
Total score	68.9 (1.7)	67.9 (1.8)	−1.0 (2.4)	.687	0.10

Notes

BT = Befriending Therapy; SCIT = Social Cognition Interaction Training; *SE* = standard error.

^a^ The reference group is BT.

**Table A2:** Antipsychotic medications by the intervention group

Type of antipsychotic	BT		SCIT		Total	
	*n*	%	*n*	%	*n*	%
Amisulpride	6	6.7	5	6.0	11	6.3
Aripiprazole	15	16.7	15	18.1	30	17.3
Asenapine	1	1.1	0	0.0	1	0.6
Clozapine	25	27.8	24	28.9	49	28.3
Flupenthixol	6	6.7	2	2.4	8	4.6
Fluphenazine (Modecate)	0	0.0	1	1.2	1	0.6
Lurasidone	1	1.1	0	0.0	1	0.6
Olanzapine	11	12.2	9	10.8	20	11.6
Paliperidone	6	6.7	16	19.3	22	12.7
Quetiapine	4	4.4	4	4.8	8	4.6
Risperidone	10	11.1	5	6.0	15	8.7
Zuclopenthixol (Clopixol)	4	4.4	0	0.0	4	2.3
Ziprasidone	1	1.1	2	2.4	3	1.7
Total	90	100.0	83	100.0	173	100.0

**Table A3:** Estimated treatment effect at post‐treatment (week 12) in the subsample of people with schizophrenia (*n* = 74)

Outcomes	Post‐intervention, least squares means (*SE*)		Between‐condition differences		
	BT	SCIT	Differences of least squares means (*SE*)^a^	*p*‐value	Effect size, Cohen’s *d*
I. BLERT					
Total score	14.3 (0.6)	15.3 (0.6)	0.9 (0.8)	.251	−0.28
II. Social Functioning Scale (SFS)					
Total score	100.4 (1.3)	103.2 (1.3)	2.8 (1.8)	.141	−0.36
III. Meta‐Cognition Questionnaire (MCQ)					
Total score	66.0 (2.1)	64.4 (2.1)	−1.6 (3.0)	.593	0.13
IV. Hinting Task (HT)					
Total score	16.8 (0.6)	16.7 (0.6)	−0.1 (0.8)	.941	0.03
V. Internal, Personal, and Situational Attributions Questionnaire (IPSAQ)					
Externalizing bias	1.8 (0.5)	2.0 (0.5)	0.2 (0.7)	.808	−0.07
Personalizing bias	0.7 (0.1)	0.7 (0.1)	0.0 (0.1)	.903	0.00
VI. Social Skills Performance Assessment (SSPA)					
Total score	68.0 (1.8)	68.2 (1.9)	0.2 (2.5)	.943	−0.02

Notes

BT = Befriending Therapy; SCIT = Social Cognition Interaction Training; *SE* = standard error.

^a^ The reference group is BT.

**Table A4:** Estimated treatment effect at post‐treatment (week 24) in the subsample of people with schizophrenia (*n* = 68)

Outcomes	Post‐intervention, least squares means (*SE*)		Between‐condition differences		
	BT	SCIT	Differences of least squares means (*SE*)^a^	*p*‐value	Effect size, Cohen’s *d*
I. BLERT					
Total score	14.7 (0.5)	15.2 (0.6)	0.4 (0.7)	.544	−0.16
II. Social Functioning Scale (SFS)					
Total score	104.4 (1.7)	106.2 (1.7)	1.7 (2.0)	.402	−0.18
III. Meta‐Cognition Questionnaire (MCQ)					
Total score	65.1 (2.2)	62.6 (2.2)	−2.5 (2.9)	.403	0.20
IV. Hinting Task (HT)					
Total score	17.2 (0.4)	17.0 (0.4)	−0.2 (0.7)	.782	0.09
V. Internal, Personal, and Situational Attributions Questionnaire (IPSAQ)					
Externalizing bias	0.7 (0.7)	1.3 (0.6)	0.6 (0.9)	.537	−0.16
Personalizing bias	0.8 (0.1)	0.9 (0.1)	0.1 (0.1)	.433	−0.17
VI. Social Skills Performance Assessment (SSPA)					
Total score	68.6 (1.3)	70.2 (1.4)	0.7 (1.9)	.730	−0.21

Notes

BT = Befriending Therapy; SCIT = Social Cognition Interaction Training; *SE* = standard error.

^a^ The reference group is BT.

**Table A5:** Estimated treatment effect by number of sessions attended at post‐treatment (week 12)

Number of sessions attended	BLERT total scores at post‐treatment (week 12)			
	Post‐intervention, least squares means (*SE*)		Between‐condition differences	
	BT	SCIT	Differences of least squares means (*SE*)^a^	*p*‐value
1–3	14.5 (1.1)	16.0 (1.0)	1.5 (1.4)	.309
4–6	15.3 (1.4)	14.6 (1.2)	−0.7 (1.9)	.733
7–9	14.2 (1.0)	14.7 (1.0)	0.5 (0.8)	.527
10–12	15.0 (0.8)	16.2 (0.9)	1.3 (1.1)	.272

Notes

^a^ The reference group is BT.
